# Editorial: Advanced Neuroimaging Methods for Studying Autism Disorder

**DOI:** 10.3389/fnins.2017.00533

**Published:** 2017-09-26

**Authors:** Alessandro Grecucci, Roma Siugzdaite, Remo Job

**Affiliations:** ^1^Clinical and Affective Neuroscience Lab (CLIAN Lab), Department of Psychology and Cognitive Sciences, University of Trento, Trento, Italy; ^2^Department of Experimental Psychology, Faculty of Psychology and Educational Sciences, Ghent University, Ghent, Belgium

**Keywords:** autism, neuroscience method, neuroimaging, fMRI, functional connectivity

Autism spectrum disorder (ASD) is a pervasive developmental disorder that affects 1 in 68 children (Christensen et al., 2016), and whose causes are still mostly unknown. Autistic symptomatology is characterized by impairments in social interaction, communication, and emotional abilities, while sparing basic cognitive skills. Many attempts have been made to provide neurobiological models of autism. Functional, structural, and connectivity analyses based on magnetic resonance imaging data have highlighted reduced responses in key social areas, such as amygdala, medial prefrontal cortex, cingulate cortex, and superior temporal sulcus. However, these studies present discrepant results and some of them have been questioned for methodological limitations. During the last few years, new neuroimaging methodologies have been developed providing more sophisticated techniques and more precise methods for investigating brain structure and function.

The aim of this research topic is to present advanced neuroimaging methods able to capture the complexity of the neural deficits displayed in autism. We present new studies using structural and functional MRI, as well as Magnetoencephalography, and novel protocols to analyze data (Analysis of Cluster Variability, Noise Reduction Strategies, Source-based Morphometry, Functional Connectivity Density, Restriction Spectrum Imaging and others). Understanding the main differences between patients and controls is of fundamental importance in at least four aspects. First, to help scholars develop more comprehensive models of autism. Second, to improve the diagnosis of autism based on objective neural markers rather than on subjective behavioral measures. Third, to facilitate early diagnosis of ASD, following clinical observations according to which the earlier the diagnosis, the better is the outcome of interventions. Fourth, a better knowledge of the neural mechanism of autism can refine and even create new treatment protocols to help these individuals. The theories and methods for studying autism presented in this state-of-the-art research topic are strongly grounded in affective neuroscience and bring together scientists describing new ways to understand the developmental pathology with innovative neuroimaging protocols and fresh ideas on the problems of diagnosis and intervention.

The issue starts with two methodological papers. Vidal et al. explore the possibility of using the Analysis of Cluster Variability to identify alterations in clustering structure of functional brain networks, and, through this method, they are able to show an atypical organization of domain-specific functional brain modules in ASD. Jann et al. evaluate the effectiveness of different noise strategies to improve perfusion-based connectivity analyses, suggesting that the removal of physiological noise and motion parameters is critical for detecting altered connectivity in neurodevelopmental disorders such as ASD.

Two morphometric studies explore the possibility of structural differences in ASD individuals. Eilam-Stock et al. apply Voxel-based Morphometry to a large sample of ASD children, trying to overcome the limitations of previous studies that used smaller samples. Decreased gray matter volume in posterior brain regions, as well as increased gray matter volume in frontal brain regions, were found in individuals with ASD. Building on the limitations of univariate approaches to morphological analyses, Grecucci et al. applied for the first time a multivariate whole brain approach known as Source-based Morphometry (SBM). This method was used on ASD individuals and controls to detect maximally independent networks of gray matter. Group comparisons revealed a network comprising broad temporal and frontal regions differently expressed in ASD individuals that correlated with social and behavioral deficits.

Alterations in brain connectivity are explored in two papers. Chen et al. used a network logic to identify abnormal functional connectivity of resting state fMRI in ASD individuals. In another connectivity study, Lee et al. decompose the inter- and intra-hemispheric regions and compare the functional connectivity density (FCD) between ASD and controls, finding evidence of FCD decreases in subjects with ASD in the posterior cingulate cortex, lingual/parahippocampal gyrus, and postcentral gyrus.

Magnetoencephalography (MEG) has been used to find cortical activation differences in ASD individuals in two studies. Khan et al. applied a novel method that measured the spatio-temporal divergence of cortical activation. It was found that the ASD group, relative to controls, is characterized by an increase in the onset component of the cortical response, and a faster spread of local activity. In an attempt to integrate fMRI with Magnetoencephalography (MEG), Datko et al. explored the links between sources of MEG amplitude in various frequency bands and functional connectivity in resting state fMRI. Hypoconnectivity between many sources of low and high gamma activity was found. This may pave the way to study differences in functionally defined networks. These studies confirm and extend results using Electroencephalography (Murias et al., 2007; Coben et al., 2014; Boutros et al., 2015; Shou et al., 2017).

One of the main practical problems clinicians are faced with is the use of objective markers to diagnose autism. Three papers make relevant contributions to this problem. A useful approach that looks for informative biomarkers of pathology in the brain is a multivariate analysis techniques based on Support Vector Machines that has been explored by Retico et al. The authors used the One-Class Classification (OCC), a reliable method that could be used as a diagnostic tool looking at language and default mode network regions that contribute most to distinguishing individuals with ASD from controls. Carper et al. used for the first time Restriction Spectrum Imaging (RSI), a multi-shell diffusion-weighted imaging technique, to examine gray matter microstructure in ASD individuals and controls, making multi-shell diffusion imaging a promising technique to understand the underlying cytoarchitecture of ASD. Last but not least, Simas and Suckling in a short commentary discuss a graph theory approach, specifically a semi-metric analysis of the functional connectome that is both sensitive and specific to psychopathologies. This suggests that resting state data are a valuable measure on which several network connectivity analysis methods can be easily applied.

On the important issue of intervention, the paper by Sperdin and Schaer reviews the critical role of *orienting to speech* in ASD, as well as the neural substrates of human voice processing, and claim that aberrant voice processing could be a promising marker to identify ASD very early on. Calderoni et al. review the neural circuit modifications after non-pharmacological interventions and stress the importance of MRI evaluation for the detection of neural changes in response to treatment.

## Conclusions and further considerations

The past 20 years witnessed a dramatic increase in the number of studies trying to uncover the pathophysiology of ASD. If it is true that neuroscience provided several proofs of abnormalities involved in autism, it is also true that this scientific endeavor failed in creating a coherent and clear picture of autism biology, so that the etiology of autism remains nowadays elusive. We suggest that in order to make progresses on this issue we need to (1) build explicit pathophysiologic models, (2) use advanced neuroimaging methods based on a whole brain and multivariate approaches; (3) integrate different neuroscientific methods (as well as other methodologies such as genetics, computational models, and other). About the first point, we believe that the practice of gathering new data not driven by explicit and testable models will not lead to a clear understanding of autism and will leave the field even more confused. Explicit pathological models are necessary to narrow down the number of factors to be taken into account. Computational methods like machine learning can find specific cerebral patterns for the disorder and classify them. For the second point, it is now clear that using a region of interest approach may obscure the importance of complex distributed networks. This is especially true for complex neuropsychiatric disorders such as autism. Third, we believe that every methodology is partial. We need to integrate data provided by different techniques in order to have a better understanding of how the brain creates autistic behavioral symptoms, and to increase the pace of a comprehensive view of autism.

## Author contributions

AG wrote the editorial, RS and RJ significantly contributed to it.

### Conflict of interest statement

The authors declare that the research was conducted in the absence of any commercial or financial relationships that could be construed as a potential conflict of interest.
